# Can Offline Testing of Deep Neural Networks Replace Their Online Testing? A Case Study of Automated Driving Systems

**DOI:** 10.1007/s10664-021-09982-4

**Published:** 2021-07-05

**Authors:** Fitash Ul Haq, Donghwan Shin, Shiva Nejati, Lionel Briand

**Affiliations:** 1 SnT, University of Luxembourg, Luxembourg City, Luxembourg; 2 University of Ottawa, Ottawa, Canada

**Keywords:** Deep Learning, Testing, Self-driving Cars

## Abstract

We distinguish two general modes of testing for Deep Neural Networks (DNNs): Offline testing where DNNs are tested as individual units based on test datasets obtained without involving the DNNs under test, and online testing where DNNs are embedded into a specific application environment and tested in a closed-loop mode in interaction with the application environment. Typically, DNNs are subjected to both types of testing during their development life cycle where offline testing is applied immediately after DNN training and online testing follows after offline testing and once a DNN is deployed within a specific application environment. In this paper, we study the relationship between offline and online testing. Our goal is to determine *how offline testing and online testing differ or complement one another* and *if offline testing results can be used to help reduce the cost of online testing?* Though these questions are generally relevant to all autonomous systems, we study them in the context of automated driving systems where, as study subjects, we use DNNs automating end-to-end controls of steering functions of self-driving vehicles. Our results show that offline testing is less effective than online testing as many safety violations identified by online testing could not be identified by offline testing, while large prediction errors generated by offline testing always led to severe safety violations detectable by online testing. Further, we cannot exploit offline testing results to reduce the cost of online testing in practice since we are not able to identify specific situations where offline testing could be as accurate as online testing in identifying safety requirement violations.

## Introduction

Deep Neural Networks (DNNs) have been widely adopted in many real-world applications, such as image classification (Ciresan et al. 2012), natural language processing (Sutskever et al. 2014), and speech recognition (Deng et al. 2013). Recent successes of DNNs on such practical problems make them key enablers of smart and autonomous systems such as automated-driving vehicles. As DNNs are increasingly used in safety critical autonomous systems, the challenge of ensuring safety and reliability of DNN-based systems emerges as a fundamental software verification problem.

**Fig. 1:**
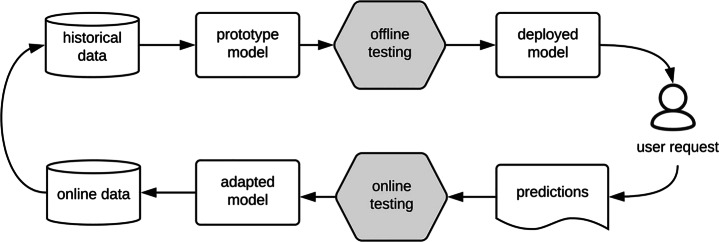
Idealized Workflow of ML testing (Zhang et al. 2020)

A main distinction between DNN testing (or in general, testing Machine Learning components) and traditional software testing is that the process of DNN testing follows a specific workflow that involves two testing phases, i.e., *offline testing* and *online testing*, as shown in Fig. 1. Offline testing is a necessary and standard step in developing Machine Learning (ML) models and is applied immediately after training a DNN model. It is used to ensure that the trained DNN model is sufficiently accurate when applied to new data (i.e., test data). Online testing, in contrast, is performed after deploying a DNN into a specific application (e.g., an automated driving system) and evaluates DNN interactions with the application environment and users. Specifically, in offline testing, DNNs are tested as a unit in an open-loop mode. They are fed with test inputs generated without involving the DNN under test, either manually or automatically. The outputs of DNNs are then typically evaluated by assessing their prediction error, which is the difference between the expected test outputs (i.e., test oracles) and the outputs generated by the DNN under test. In online testing, however, DNNs are tested within an application environment in a closed-loop mode. They receive test inputs generated by the environment, and their outputs are, then, directly fed back into the environment. Online testing evaluates DNNs by monitoring the requirements violations they trigger, for example related to safety. Given the safety critical nature of many systems relying on DNNs (e.g., self-driving cars), most online testing approaches rely on simulators, as testing DNNs embedded into real and operational environment is expensive, time consuming and often dangerous.

Recently, many DNN testing techniques and algorithms have been proposed in the literature (Zhang et al. 2020). However, the majority of the existing DNN testing techniques are either specifically designed for offline testing or even if they could be applied in an online setting, they are still solely evaluated and assessed in an offline setting. This is partly because offline testing matches the standard checking of an ML model in terms of prediction accuracy, that does not require the DNN to be embedded into an application environment and can be readily carried out with either manually generated or automatically generated test data. Given the increasing availability of open-source data, a large part of offline testing research uses open-source, manually-generated real-life test data. Online testing, on the other hand, necessitates embedding a DNN into an application environment, either real or simulated.

Even though online testing is less studied, it remains an important phase for DNN testing for a number of reasons (Zhang et al. 2020). First, the test data used for offline testing may not be representative of the data that a DNN should eventually be able to handle when it is embedded into a specific application. Second, in contrast to offline testing, online testing is able to assess the DNN interactions with an application environment and can reveal failures that can only occur with real applied scenarios (e.g., if an accident actually happens in self-driving cars on real driving scenarios). In other words, while offline testing results are limited to assessing prediction errors or prediction accuracy, online testing results can be used to directly assess system-level requirements (e.g., whether or not an accident happened, or if there is a security breach, or if there is a data loss or communication error).

At a high-level, we expect offline testing to be faster and less expensive than online testing because offline testing does not require a closed-loop environment to generate test inputs. However, there is limited insight as to how these two testing modes compare with one another with respect to their ability to reveal faulty behaviors and most particularly those leading to safety violations. This, for example, would depend if large prediction errors identified by offline testing always lead to safety violations detectable by online testing, or if the safety violations identified by online testing translate into large prediction errors. Answers to these questions would enable us to better understand the limitations of the two testing stages and their relationship.

We investigated the above questions in an empirical study and presented the results in a conference paper (Haq et al. 2020a) published in International Conference on Software Testing, Verification and Validation (ICST 2020). Though the investigated questions are generally relevant to all autonomous systems, we performed an empirical study to compare offline testing and online testing in the context of Automated Driving Systems (ADS). In particular, our study aimed to ultimately answer the following research question: *How do offline and online testing results differ and complement each other?* To answer this question, we used open-source DNN models developed to automate steering functions of self-driving vehicles (Udacity 2016a). To enable online testing of these DNNs, we integrated them into a powerful, high-fidelity physics-based simulator of self-driving cars (TASS International - Siemens Group 2019). The simulator allows us to specify and execute scenarios capturing various road traffic situations, different pedestrian-to-vehicle and vehicle-to-vehicle interactions, and different road topologies, weather conditions and infrastructures. As a result, in our study offline and online testing approaches were compared with respect to the data generated automatically using a simulator. To ensure that this aspect does not impact the validity of our comparison, we investigated the following research question as a pre-requisite of the above question: *Can we use simulator-generated data as a reliable substitute to real-world data for the purpose of DNN testing?*

While the above research questions provide insights on the relationship between offline and online testing results, it is still unclear how we can use offline and online testing together in practice such that we can minimize cost and maximize the effectiveness of testing DNNs. As the ML testing workflow in Fig. 1 suggests, offline testing always precedes online testing and given that offline testing is considerably less expensive than online testing, it is beneficial if we can exploit offline testing results to reduce the cost of online testing by running fewer tests. In this article, we introduce a new research question to determine *if offline testing results can be used to help reduce the cost of online testing?* Our goal is to identify whether we can characterize the test scenarios (conditions) where offline and online testing results are the same with high probability. To do so, we propose a novel heuristic approach to infer such conditions from limited number of offline and online testing data in an efficient and effective way.

The contributions of this article are summarized below:

1. We show that we can use simulator-generated datasets in lieu of real-life datasets for testing DNNs in our application context. Our comparison between online and offline testing using such datasets show that offline and online testing results frequently differ, and specifically, offline testing results are often not able to find faulty behaviors due to the lack of error accumulation over time. As a result, many safety violations identified by online testing could not be identified by offline testing as they did not cause large prediction errors. However, all the large prediction errors generated by offline testing led to severe safety violations detectable by online testing.
2. We provide a three-step approach to infer (learn) conditions characterizing agreement and disagreement between offline and online testing results while minimizing the amount of the data required to infer the conditions and maximizing the statistical confidence of the results.
3. We were not able to infer any conditions that can characterize agreement between offline and online testing results with a probability higher than 71%. This means that, in general, we cannot exploit offline testing results to reduce the cost of online testing in practice.

The first contribution is mainly the result of our first two research questions presented in our earlier work (Haq et al. 2020a). In this article, we have, however, extended our first contribution in two ways: *First,* our earlier work used two ADS DNNs from the Udacity challenge (Udacity 2016a), namely Autumn and Chauffeur. In this article, we add another DNN model (Komanda) from the same Udacity challenge to the set of our study subjects to strengthen our results. We also surveyed other ADS-DNNs from the Udacity challenge and other sources, but we were not able to find any other suitable study subject candidate since other public ADS-DNNs are either significantly more inaccurate (higher prediction errors) than our three selected DNNs or their inputs and outputs were not compatible with our simulator, and hence, we could not test them in an online setting. *Second,* in this article, we provide additional correlation analysis between offline and online testing to support our results.

The second and third contributions are completely new and have not been presented before. In addition, in this article, we refine and extend ideas from our previous work and extend our discussion of the related literature. Through our experiments, we collected both offline and online testing results for more than 700 test scenarios in total, taking around 350 hours of simulations, resulting in around 50 GB of simulator-generated images. To facilitate the replication of our study, we have made all the experimental materials, including simulator-generated data, publicly available (Haq et al. 2020b).

The rest of the paper is organized as follows: Section 2 provides background on DNNs for autonomous vehicles, introduces offline and online testing, describes our proposed domain model that is used to configure simulation scenarios for automated driving systems, and formalizes the main concepts in offline and online testing used in our experiments. Section 3 reports on the empirical evaluation. Section 5 surveys the existing research on online and offline testing for automated driving system. Section 6 concludes the paper.

## Background

This section provides the basic concepts that will be used throughout the article.

### DNNs in ADS

**Fig. 2:**
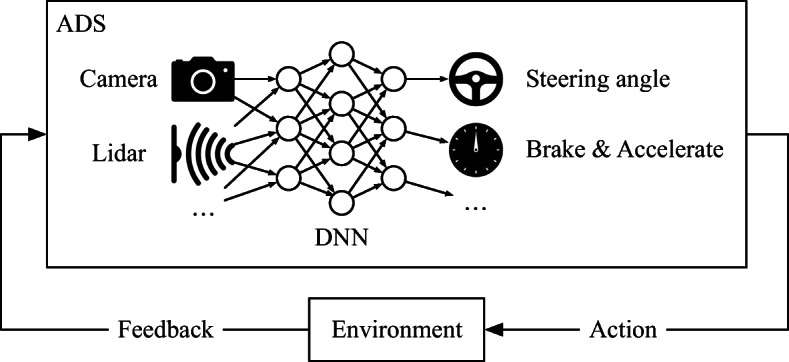
Overview of DNN-based ADS

Depending on the ADS design, DNNs may be used in two ways to automate the driving task of a vehicle: One design approach is to incorporate DNNs into the ADS perception layer, primarily to do *semantic segmentation* (Geiger et al. 2012), i.e., to classify and label each and every pixel in a given image. The ADS software controller then decides what commands should be issued to the vehicle’s actuators based on the classification results produced by the DNN (Pomerleau 1989). An alternative design approach is to use DNNs to perform the *end-to-end* control of a vehicle (Udacity 2016a) (e.g., Fig. 2). In this case, DNNs directly generate the commands to be sent to the vehicle’s actuators after processing images received from cameras. Our approach to compare offline and online testing of DNNs is applicable to both ADS designs. In the comparison provided in this article, however, we use DNN models automating the end-to-end control of the steering function since these models are publicly available online and have been extensively used in recent studies on DNN testing (Tian et al. 2018; Zhang et al. 2018; Ma et al. 2018; Kim et al. 2019). In particular, we investigate the DNN models from the Udacity self-driving challenge as our study subjects (Udacity 2016a). We refer to this class of DNNs as ADS-DNNs in the remainder of the article. Specifically, an ADS-DNN receives as input images from a front-facing camera mounted on a vehicle, and generates a steering angle command for the vehicle.

### Test Data Sources

We identify two sources for generating test data for testing ADS-DNNs: (1) real-life driving and (2) driving simulator.

For our ADS-DNN models, a *real-life dataset* is a video or a sequence of images captured by a camera mounted on a vehicle’s dashboard while the vehicle is being driven by a human driver. The steering angle of the vehicle applied by the human driver is recorded for the duration of the video and each image (frame) of the video in this sequence is labelled by its corresponding steering angle. This yields a sequence of manually labelled images to be used for testing DNNs. There are, however, some drawbacks with test datasets captured from real-life (Kalra and Paddock 2016). Specifically, data generation is expensive, time consuming and lacks diversity. The latter issue is particularly critical since driving scenes, driving habits, as well as objects, infrastructures and roads in driving scenes, can vary widely across countries, continents, climates, seasons, day times, and even drivers.

Another source of test data generation is to use simulators to automatically generate videos capturing various driving scenarios. There are increasingly more high-fidelity and advanced physics-based simulators for self-driving vehicles fostered by the needs of the automotive industry, which increasingly relies on simulators to improve their testing and verification practices. There are several examples of commercial ADS simulators (e.g., PreScan (TASS International - Siemens Group 2019) and Pro-SiVIC (ESI Group 2019)) and a number of open source ones (e.g., CARLA (Dosovitskiy et al. 2017) and LGSVL (Rong et al. 2020)). These simulators incorporate dynamic models of vehicles (including vehicles’ actuators, sensors and cameras) and humans as well as various environment aspects (e.g., weather conditions, different road types, different infrastructures). The simulators are highly configurable and can be used to generate desired driving scenarios. In our work, we use the PreScan simulator to generate test data for ADS-DNNs. PreScan is a widely-used, high-fidelity commercial ADS simulator in the automotive domain and has been used by our industrial partner. In Section 2.3, we present the domain model that define the inputs used to configure the simulator, and describe how we automatically generate scenarios that can be used to test ADS-DNNs. Similar to real-life videos, the videos generated by our simulator are sequences of labelled images such that each image is labelled by a steering angle. In contrast to real-life videos, the steering angles generated by the simulator are automatically computed based on the road trajectory as opposed to being generated by a human driver.

The simulator-generated test datasets are cheaper and faster to produce compared to real-life ones. In addition, depending on how advanced and comprehensive the simulator is, we can achieve a higher-level of diversity in the simulator-generated datasets by controlling and varying the objects, roads, weather, and other various features. However, it is not yet clear whether simulator-generated images can be used in lieu of real images since the latter may have higher resolution, showing more natural texture, and look more realistic. In this article, we conduct an empirical study in Section 3 to investigate *if we can use simulator-generated images as a reliable alternative to real images for testing ADS-DNNs*.

### Domain Model

**Fig. 3:**
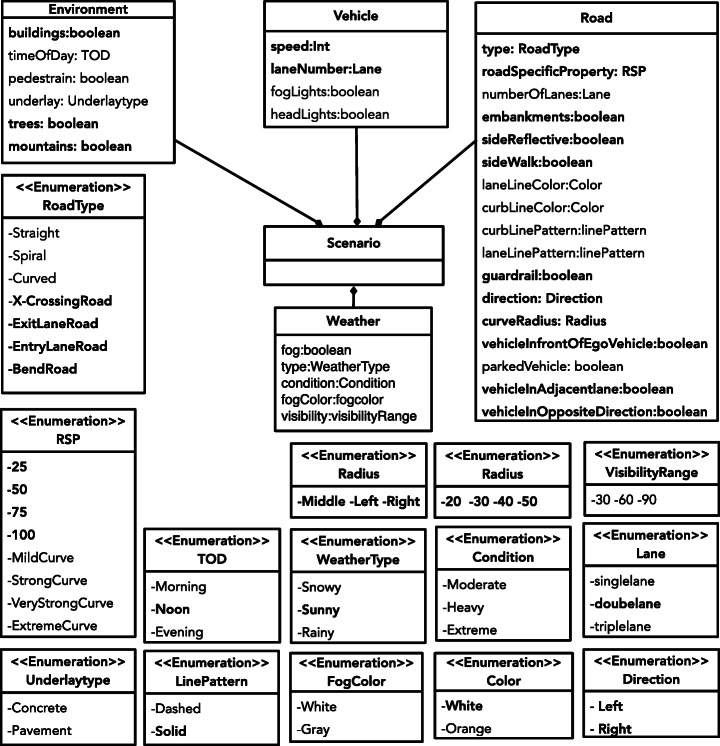
Complete domain model for scenario generation. The attributes and values that are observed in the real-world test datasets are highlight in bold

Figure 3 shows the domain model capturing the test input space of ADS-DNNs. To develop the domain model, we relied on two sources of information: (1) the properties that we observed in the real-world ADS-DNN test datasets (i.e., the Udacity testing datasets (Udacity 2016b)) and (2) the configurable parameters of our simulator. In total, we identified four main objects, i.e., *Road*, *Vehicle*, *Weather*, and *Environment*, and 32 attributes characterizing them, such as *Road.type*, *Vehicle.speed*, *Weather.type*, and *Environment.buildings*. Each attribute has a specific data type; for example, the *Weather.type* attribute is an enumeration type, having three different weather values (i.e., *Snowy*, *Sunny*, and *Rainy*) as shown in the definition of *Weather.Type* in Fig. 3. This means that only one of the three values can be assigned to *Weather.type*. Note that, to illustrate the lower diversity in real-world datasets, the attributes and their values that are observed in the real world are highlighted in bold. For example, only the *Sunny* weather is observed in the real-world test datasets.

In addition to objects and attributes, our domain model includes some constraints describing valid value assignments to the attributes. These constraints mostly capture the physical limitations and traffic rules that apply to our objects. For example, the vehicle speed cannot be higher than 20km/h on steep curved roads. Constraints may also be used to capture dependencies between attributes that cannot be specified through the relationships between domain model objects. For example, we define a constraint to indicate that *Weather.condition* can only take a value when *Weather.type* is either *Snowy* or *Rainy*. That is, for *Sunny* we do not need to specify any weather condition. We have specified these constraints in the Object Constraint Language (OCL) (Group 2014). The complete OCL constraints are available in the supporting materials (Haq et al. 2020b).

To produce a simulation scenario (or test scenario) for an ADS-DNN, we instantiate our domain model in Fig. 3 by assigning concrete values to the attributes of our domain model such that its OCL constraints are satisfied. Specifically, we can represent each test scenario as a vector $\mathbf {s} = \langle v_{1}, v_{2}, \dots , v_{32} \rangle $ where *v*_*i*_ is the value assigned to the *i* th attribute of our domain model (recall that it contains 32 attributes). We can then initialize the simulator based on the test scenario vectors. The simulator will then generate, for each of the mobile objects defined in a scenario, namely the ego and secondary vehicles and pedestrians, a trajectory vector of the path of that object (i.e., a vector of values indicating the positions and speeds of the mobile object over time). The length of the trajectory vector is determined by the duration of the simulation. The position values are computed based the characteristics of the static objects specified by the initial configuration, such as roads and sidewalks, as well as the speed of the mobile objects.

### Offline Testing

**Fig. 4:**
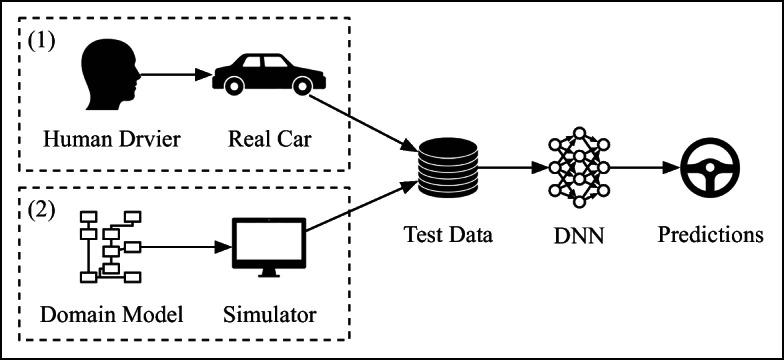
Offline testing using (1) real-world and (2) simulator-generated data

Figure 4 represents an overview of offline DNN testing in the ADS context. Briefly, offline testing verifies the DNN using historical data consisting of sequences of images captured from real-life camera or based on a camera model of a simulator. In either case, the images are labelled with steering angles. Offline testing measures the *prediction errors* of the DNN to evaluate test results.

More specifically, let **r** be a real-life test dataset composed of a sequence of tuples $\langle ({i}_{1}^{r}, {\theta }_{1}^{r}), ({i}_{2}^{r}, {\theta }_{2}^{r}), \dots , ({i}_{n}^{r}, {\theta }_{n}^{r}) \rangle $. For $j=1,\dots ,n$, each tuple $({i}_{j}^{r}, {\theta }_{j}^{r})$ of **r** consists of an image ${i^{r}_{j}}$ and a steering angle ${\theta ^{r}_{j}}$ label. A DNN *d*, when provided with a sequence $\langle {i}_{1}^{r}, {i}_{2}^{r}, \dots , {i}_{n}^{r} \rangle $ of the images of **r**, returns a sequence $\langle \hat {\theta }_{1}^{r}, \hat {\theta }_{2}^{r}, \dots , \hat {\theta }_{n}^{r} \rangle $ of predicted steering angles. The prediction error of *d* for **r** is, then, computed using two well-known metrics, Mean Absolute Error (MAE) and Root Mean Square Error (RMSE), defined below:

$$ \begin{array}{@{}rcl@{}} {MAE}(d, \mathbf{r}) &= \frac{{\sum}_{i=1}^{n} |{\theta}_{i}^{r} - \hat{{\theta}_{i}^{r}}|}{n}\\ {RMSE}(d, \mathbf{r}) &= \sqrt{\frac{{\sum}_{i=1}^{n} ({\theta}_{i}^{r} - \hat{{\theta}_{i}^{r}})^{2}}{n}} \end{array} $$

To generate a test dataset using a simulator, we provide the simulator with an initial configuration of a scenario as defined in Section 2.3. We denote the offline test dataset generated by a simulator for a scenario **s** by ${sim}(\mathbf {s}) = \langle ({i}_{1}^{s}, {\theta }_{1}^{s}), ({i}_{2}^{s}, {\theta }_{2}^{s}), \dots , ({i}_{n}^{s}, {\theta }_{n}^{s}) \rangle $. The prediction error of *d* for *sim*(**s**) is calculated by the MAE and RMSE metrics in the same way as *MAE*(*d*,**r**) and *RMSE*(*d*,**r**), replacing **r** with *sim*(**s**).

### Online Testing

**Fig. 5:**
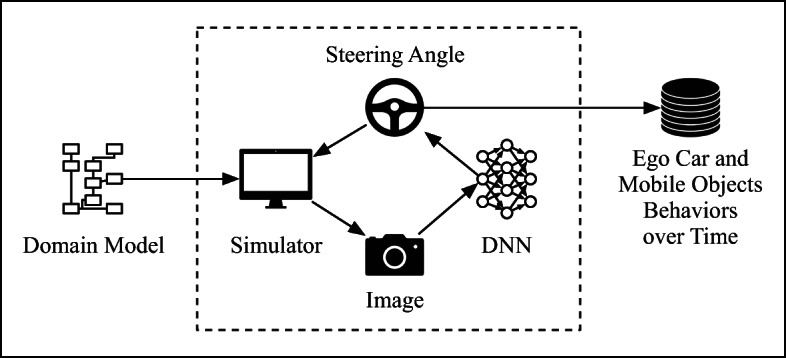
Online testing of ADS-DNNs using simulators

Figure 5 provides an overview of online testing of DNNs in the ADS context. In contrast to offline testing, DNNs are embedded into a driving environment, often in a simulator due to the cost and risk of real-world testing as we described in Section 2.2. DNNs then receive images generated by the simulator, and their outputs are directly sent to the (ego) vehicle models of the simulator. With online testing, we can evaluate how predictions generated by an ADS-DNN, for an image generated at time *t* in a scenario, impact the images to be generated at the time steps after *t*. In addition to the steering angle outputs directly generated by the ADS-DNN, we obtain the trajectory outputs of the ego vehicle, which enable us to determine whether the vehicle is able to stay in its lane.

More specifically, we embed a DNN *d* into a simulator and run the simulator. For each (initial configuration of a) scenario, we execute the simulator for a time duration *T*. The simulator generates the trajectories of mobile objects as well as images taken from the front-facing camera of an ego vehicle at regular time steps *t*_*δ*_, generating outputs as vectors of size $m=\lfloor \frac {T}{t_{\delta }} \rfloor $. Each simulator output and image takes an index between 1 to *m*. We refer to the indices as simulation time steps. At each time step *j*, the simulator generates an image ${i^{s}_{j}}$ to be sent to *d* as input, and *d* predicts a steering angle $\hat {\theta }^{s}_{j}$ which is sent to the simulator. The status of the ego vehicle is then updated in the next time step *j* + 1 (i.e., the time duration it takes to update the vehicle is *t*_*δ*_) before the next image $i^{s}_{j+1}$ is generated. In addition to images, the simulator generates the position of the ego vehicle over time. Recall that the main function of our DNN is automated lane keeping. This function is violated when the ego vehicle departs from its lane. To measure the lane departure degree, we use the Maximum Distance from Center of Lane (MDCL) metric for the ego vehicle to determine if a safety violation has occurred. The value of MDCL is computed at the end of the simulation when we have the position vector of the ego vehicle over time steps, which was guided by our DNN. We cap the value of MDCL at 1.5 m, indicating that when MDCL is 1.5 m or larger, the ego vehicle has already departed its lane and a safety violation has occurred. In addition, we normalize the MDCL values between 0 and 1 to make it consistent with MAE or RMSE.

In this article, we embed the ADS-DNN into PreScan by providing the former with the outputs from the camera model in input and connecting the steering angle output of the ADS-DNN to the input command of the vehicle dynamic model.

## Experiments

We aim to compare offline and online testing of DNNs by answering the following research questions:

**RQ1:** Can we use simulator-generated data as a reliable alternative source to real-world data? Recall the two sources for generating test data as described in Section 2.2. While simulator-generated test data is cheaper and faster and is more amenable to input diversification compared to real-life test data, the texture and resolution of real-life data look more natural and realistic compared to the simulator-generated data. In RQ1, we aim to investigate whether, or not, such differences lead to significant inaccuracies in predictions of the DNN under test in offline testing. The answer to this question will determine if we can rely on simulator-generated data for testing DNNs in either offline or online testing modes.

**RQ2:** How frequently do offline and online testing results differ and do they complement each other? RQ2 is one of the main research questions we want to answer in this paper. We want to know how the results obtained by testing a DNN in isolation, irrespective of a particular application context, compare with the results obtained by embedding a DNN into a specific application environment. The answer to this question will help engineers and researchers better understand the applications and limitations of each testing mode, and how they could possibly be combined.

**RQ3:** Can offline testing results be used to help reduce the cost of online testing? In other words, can we focus online testing on situations where it is needed, i.e., on situations where offline and online testing are in disagreement? With RQ3, we investigate whether any offline testing results can be lifted to online testing to help reduce the amount of online testing that we need to do. Our goal is to determine whether we can characterize the test scenarios where offline and online testing behave the same in terms of our domain model elements. This provides the conditions under which offline testing is sufficient, thus avoiding online testing, which is much more expensive.

### Experimental Subjects

We use three publicly-available, pre-trained DNN-based steering angle prediction models, i.e., Autumn (2016), Chauffeur (2016), and (Komanda 2016), that have been widely used in previous work to evaluate various DNN testing approaches (Tian et al. 2018; Zhang et al. 2018; Kim et al. 2019).

Autumn consists of an image preprocessing module implemented using OpenCV to compute the optical flow of raw images, and a Convolutional Neural Network (CNN) implemented using Tensorflow and Keras to predict steering angles. Autumn improved performance by using cropped images from the bottom half of the entire images. Chauffeur consists of one CNN that extracts the features from raw images and a Recurrent Neural Network (RNN) that predicts steering angles from the previous 100 consecutive images with the aid of a LSTM (Long Short-Term Memory) module. Similar to Autumn, Chauffeur uses cropped images, and is also implemented with Tensorflow and Keras. Komanda consists of one CNN followed by one RNN with LSTM, implemented by Tensorflow, similar to Chauffeur. However, the underlying CNN of Komanda has one more dimension than Chauffeur that is in charge of learning spatiotemporal features. Further, unlike Autumn and Chauffeur, Komanda uses full images to predict steering angles.

**Fig. 6:**
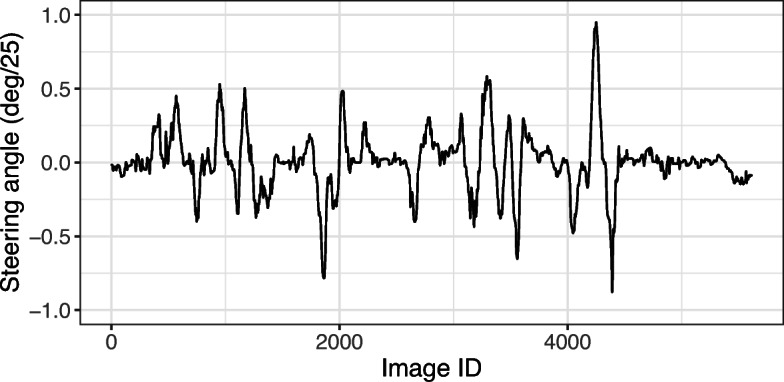
Actual steering angles for the 5614 real-world images used for testing

The models are developed using the Udacity dataset (2016b), which contains 33808 images for training and 5614 images for testing. The images are sequences of frames of two separate videos, one for training and one for testing, recorded by a dashboard camera with 20 Frame-Per-Second (FPS). The dataset also provides, for each image, the actual steering angle produced by a human driver while the videos were recorded. A positive (+) steering angle represents turning right, a negative (-) steering angle represents turning left, and a zero angle represents staying on a straight line. The steering angle values are normalized (i.e., they are between − 1 and + 1) where a + 1 steering angle value indicates 25^∘^, and a − 1 steering angle value indicates -25^∘^^1^. Figure 6 shows the actual steering angle values for the sequence of 5614 images in the test dataset. We note that the order of images in the training and test datasets matters and is accounted for when applying the DNN models. As shown in the figure, the steering angles issued by the driver vary considerably over time. The large steering angle values (more than 3^∘^) indicate actual road curves, while the smaller fluctuations are due to the natural behavior of the human driver even when the vehicle drives on a straight road.

Table 1 shows the RMSE and MAE values of the two models we obtained for the Udacity test dataset, as well as the RMSE values reported by the Udacity website (2016a)^2^. The differences are attributed to challenges regarding reproducibility, a well-known problem for state-of-the-art deep learning methods (Pineau 2019) because they involve many parameters and details whose variations may lead to different results. Specifically, even though we tried to carefully follow the same settings and parameters as those suggested on the Udacity website, as shown in Table 1, the RMSE and MAE values that we computed differed from those reported by Udacity. We believe these differences are due to the versions of python and other required libraries (e.g., tensorflow, keras, and scipy). The precise version information for all these were not reported by Udacity. Nevertheless, for all of our experiments, we consistently used the most stable versions of python and the libraries that were compatible with one another. In other words, irrespective of differences in the RMSE values between the reported and our in Table 1, all of our experiments are internally consistent. To enable replication of our work, we have made our detailed configurations (e.g., python and auxiliary library versions), together with supporting materials, available online (Haq et al. 2020b).

**Table 1:** Accuracies of the subject DNN-based models

Model	Reported RMSE	Our RMSE	Our MAE
Autumn	Not Presented	0.049	0.034
Chauffeur	0.058	0.092	0.055
Komanda	0.048	0.058	0.039

While MAE and RMSE are two of the most common metrics used to measure prediction errors for learning models with continuous variable outputs, we mainly use MAE throughout this article because, in contrast to RMSE, MAE values can be directly interpreted in terms of individual steering angle values. For example, *MAE*(*d*,**r**) = 1 means that the average prediction error of *d* for the images in **r** is 1 (25^∘^). Since MAE is a more intuitive metric for our purpose, we will only report MAE values in the remainder of this article.

### RQ1: Comparing Offline Testing Results for Real-life Data and Simulator-generated Data

#### Setup

We aim to generate simulator-generated datasets closely mimicking the Udacity real-life test dataset and verify whether the prediction errors obtained by applying DNNs to the simulator-generated datasets are comparable with those obtained for their corresponding real-life ones. As explained in Section 3.1, our real-life test dataset is a sequence of 5614 images labelled with their corresponding actual steering angles. If we could precisely extract the properties of the environment and the dynamics of the ego vehicle from the real-life datasets, in terms of initial configuration parameters of the simulator, we could perhaps generate simulated data closely resembling the real-life videos. However, extracting information from such video images to generate inputs of a simulator is not possible.

Instead, we propose a two-step heuristic approach to replicate the real-life dataset using our simulator. Basically, we steer the simulator to generate a sequence of images similar to the images in the real-life dataset such that the steering angles generated by the simulator are close to the steering angle labels in the real-life dataset.

In the first step, we observe the test dataset and manually identify the information in the images that correspond to some attribute values in our domain model described in Section 2.3. We then create a “restricted” domain model by fixing the attribute values in our domain model to the values we observed in the Udacity test dataset. This enables us to steer the simulator to resemble the characteristics of the images in the test dataset to the extent possible. Our restricted domain model includes the attributes and its values that are highlighted in bold in Fig. 3. For example, the restricted domain model does not include weather conditions other than sunny because the test dataset has only sunny images. This guarantees that the simulator-generated images based on the restricted domain model represent sunny scenes only. Using the restricted domain model, we randomly generate a large number of scenarios yielding a large number of simulator-generated datasets.

In the second step, we aim to ensure that the datasets generated by the simulator have similar steering angle labels as the labels in the real-life dataset. To ensure this, we match the simulator-generated datasets with (sub)sequences of the Udacity test dataset such that the similarities between their steering angles are maximized. Note that steering angle is *not* a configurable attribute in our domain model, and hence, we could not force the simulator to generate data with steering angle values identical to those in the test dataset by restricting our domain model. In other words, we minimize the differences by selecting the closest simulator-generated datasets from a large pool of randomly generated ones. To do this, we define, below, the notion of “comparability” between a real-life dataset and a simulator-generated dataset in terms of steering angles.

Let *S* be a set of randomly generated scenarios using the restricted domain model, and let $\mathbf {r} = \langle ({i^{r}_{1}}, {\theta ^{r}_{1}}), \dots , ({i^{r}_{k}}, {\theta ^{r}_{k}}) \rangle $ be the Udacity test dataset where *k* = 5614. We denote by $\mathbf {r}_{(x,l)} = \langle (i^{r}_{x+1}, \theta ^{r}_{x+1}), \dots , (i^{r}_{x+l}, \theta ^{r}_{x+l}) \rangle $ a subsequence of **r** with length *l* starting from index *x* + 1 where $x\in \{0, 1, \dots , k\}$. For a given simulator-generated dataset ${sim}(\mathbf {s}) = \langle ({i^{s}_{1}}, {\theta ^{s}_{1}}), \dots , ({i^{s}_{n}}, {\theta ^{s}_{n}}) \rangle $ corresponding to a scenario **s** ∈ *S*, we compute **r**_(*x*,*l*)_ using the following three conditions:

1 $$ \begin{array}{@{}rcl@{}} l &=& n \end{array} $$

2 $$ \begin{array}{@{}rcl@{}} x &=& \underset{x}{\text{argmin}} \sum\limits_{j=1}^{l} \left|{\theta}_{j}^{s} - {\theta}_{x+j}^{r}\right| \end{array} $$

3 $$ \begin{array}{@{}rcl@{}} &&\frac{{\sum}_{j=1}^{l} \left|{\theta}_{j}^{s} - {\theta}_{x+j}^{r}\right|}{l} \le \epsilon \end{array} $$

where argmin_*x*_*f*(*x*) returns^3^*x* minimizing *f*(*x*), and *𝜖* is a small threshold on the average steering angle difference between *sim*(**s**) and **r**_(*x*,*l*)_. We say datasets *sim*(**s**) and **r**_(*x*,*l*)_ are *comparable* if and only if **r**_(*x*,*l*)_ satisfies the three above conditions (i.e., (1), (2) and (3)).

Given the above formalization, our approach to replicate the real-life dataset **r** using our simulator can be summarized as follows: In the first step, we randomly generate a set of many scenarios *S* based on the reduced domain model. In the second step, for every scenario **s** ∈ *S*, we identify a subsequence **r**_(*x*,*l*)_ from **r** such that *sim*(**s**) and **r**_(*x*,*l*)_ are comparable.

If *𝜖* is too large, we may find that **r**_(*x*,*l*)_ has steering angles that are too different from those in *sim*(**s**). On the other hand, if *𝜖* is too small, we may not be able to find a **r**_(*x*,*l*)_ that is comparable to *sim*(**s**) for many scenarios **s** ∈ *S* randomly generated in the first step. In our experiments, we select *𝜖* = 0.1 (2.5^∘^) since, based on our preliminary evaluations, we can achieve an optimal balance with this threshold.

For each comparable pair of datasets *sim*(**s**) and **r**_(*x*,*l*)_, we measure the *prediction error difference* for the same DNN to compare the datasets. Specifically, we measure |*MAE*(*d*,*sim*(**s**)) − *MAE*(*d*,**r**_(*x*,*l*)_)| of a DNN *d*. Recall that offline testing results for a given DNN *d* are measured based on prediction errors in terms of MAE. If |*MAE*(*d*,*sim*(**s**)) − *MAE*(*d*,**r**_(*x*,*l*)_)|≤ 0.1 (meaning 2.5^∘^ of average prediction error across all images), we say that **r**_(*x*,*l*)_ and *sim*(**s**) yield *consistent* offline testing results for *d*.

We note that the real-life images in the Udacity test dataset are multicolored or polychromatic. However, our preliminary evaluation confirmed that the steering predictions of our DNN subjects do not change more than 0.006^∘^ on average when we convert polychromatic images to monochromatic images in the Udacity test dataset. Hence, we do not attempt to make the colors of the simulator-generated images similar to that of the real-life images as color has little impact on the DNN’s predictions.

#### Results

**Fig. 7:**
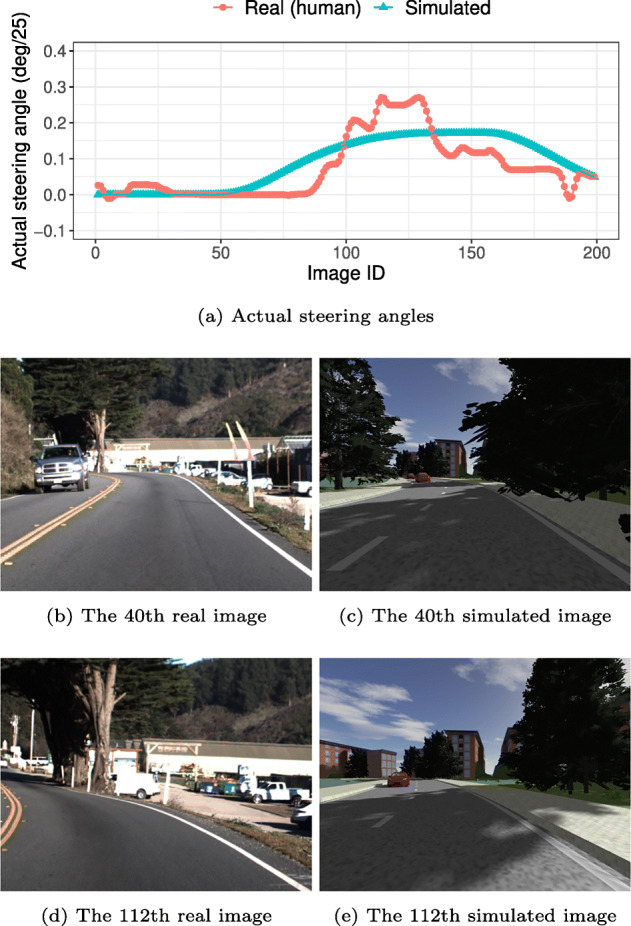
Example comparable pair of a simulator-generated and real-life datasets

Among the 100 randomly generated scenarios (i.e., |*S*| = 100), we identified 92 scenarios that could match subsequences of the Udacity real-life test dataset. Figure 7 shows an example comparable pair of **r**_(*x*,*l*)_ (i.e., real dataset) and *sim*(**s**) (i.e., simulator-generated dataset) identified using our two-step heuristic. Specifically, Fig. 7(a) shows the steering angles for all the images in the example comparable pair. Figures 7(b) and 7(c) show two matching frames from the pair where the difference in the steering angles is the smallest (i.e., the 40th frames where |*𝜃*^*r*^ − *𝜃*^*s*^| = 0). Figures 7(d) and 7(e) show two other matching frames from the pair where the difference in the steering angles is the largest (i.e., the 112th frames where |*𝜃*^*r*^ − *𝜃*^*s*^| = 0.1115). As shown in the steering angle graph in Fig. 7(a), the simulator-generated dataset and its comparable real dataset subsequence do not have identical steering angles. For example, the actual steering angles produced by a human driver have natural fluctuations whereas the steering angles generated by the simulator are relatively smooth. The differences in steering angles can also be attributed to the complexity of the real-world not reflected in the simulator (e.g., bumpy roads). Nevertheless, the overall steering angle patterns are very similar. If we look at the matching frames shown in Figs. 7(d) and 7(c), the matching frames look quite similar in terms of essential properties, such as road topology and incoming vehicles on the other lane. Regarding the matching frames shown in Figs. 7(d) and 7(e), they capture the largest difference in steering angles the comparable pair of real and simulated datasets. We can note differences between the matching frames regarding some aspects, such as the shape of buildings and trees. Once again, this is because the complexity and diversity of the real-world is not fully reflected in the simulator. This point will be further discussed in Section 4.1.

**Fig. 8:**
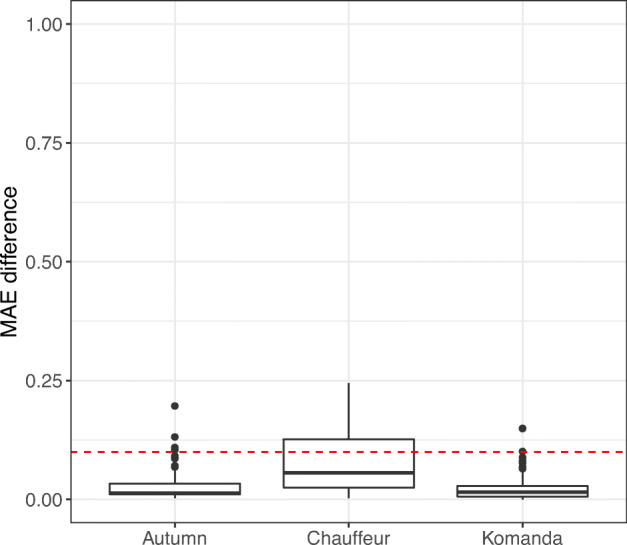
Distributions of the differences between the prediction errors obtained for the real datasets (subsequences) and the simulator-generated datasets

Figure 8 shows, for each of our DNNs, Autumn, Chauffeur, and Komanda, the distributions of the prediction error differences for the real datasets (subsequences) and the simulator-generated datasets. For Autumn, the average prediction error difference between the real datasets and the simulator-generated datasets is 0.027. Further, 95.6% of the comparable pairs show a prediction error difference below 0.1 (2.5^∘^). This means that the (offline) testing results obtained for the simulator-generated datasets are consistent with those obtained using the real-world datasets for almost all comparable dataset pairs. The results for Komanda are similar: the average prediction error difference is 0.023, and 96.7% of the comparable pairs show a prediction error difference below 0.1 (2.5^∘^). On the other hand, for Chauffeur, only 66.3% of the comparable pairs show a prediction error difference below 0.1. This means that testing results between real datasets and simulator-generated datasets are inconsistent in 33.71% of the 92 comparable pairs. Specifically, for *all* the inconsistent cases, we observed that the MAE value for the simulator-generated dataset is greater than its counterpart for the real-world dataset. It is therefore clear that the prediction error of Chauffeur tends to be larger for the simulator-generated dataset than for the real-world dataset. In other words, the simulator-generated datasets tend to be conservative for Chauffeur and report more false positives than for Autumn and Komanda in terms of prediction errors. We also found that, in several cases, Chauffeur’s prediction errors are greater than 0.2 while Autumn’s and Komanda’s prediction errors are less than 0.1 for the same simulator-generated dataset. One possible explanation is that Chauffeur is over-fitted to the texture of real images, while Autumn is not thanks to the image preprocessing module. Nevertheless, the average prediction error differences between the real datasets and the simulator-generated datasets is 0.080 for Chauffeur, which is still less than 0.1. This implies that, although Chauffeur will lead to more false positives (incorrect safety violations) than Autumn and Komanda, the number of false positives is still unlikely to be overwhelming.

We remark that the choice of simulator as well as the way we generate data using our selected simulator, based on carefully designed experiments such as the ones presented here, are of great importance. Selecting a suboptimal simulator may lead to many false positives (i.e., incorrectly identified prediction errors) rendering simulator-generated datasets ineffective. 
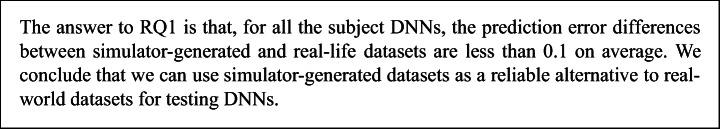


### RQ2: Comparison between Offline and Online Testing Results

#### Setup

We aim to compare offline and online testing results in this research question. We randomly generate scenarios and compare the offline and online testing results for each of the simulator-generated datasets.

For the scenario generation, we use the extended domain model (see Fig. 3) to take advantage of all the feasible attributes provided by the simulator. Specifically, in Fig. 3, the gray-colored entities and attributes in bold are additionally included in the extended domain model compared to the restricted domain model used for RQ1. For example, the (full) domain model contains various weather conditions, such as rain, snow, and fog, in addition to sunny.

Let $S^{\prime }$ be the set of randomly generated scenarios based on the (full) domain model. For each scenario $\mathbf {s}\in S^{\prime }$, we prepare the simulator-generated dataset *sim*(**s**) for offline testing and measure *MAE*(*d*,*sim*(**s**)) for a DNN *d*. For online testing, we measure *MDCL*(*d*,**s**). Then we compute the Spearman rank correlation coefficient *ρ* (rho) between *MAE*(*d*,*sim*(**s**)) and *MDCL*(*d*,**s**) to assess the overall correlation between offline and online testing results. When *ρ* is 0, it means that there is no monotonic relation between MAE and MDCL. The closer *ρ* to 1, the closer the relation between MAE and MDCL to a perfectly monotonic relation. When *ρ* is 1, it means that MAE systematically increases (decreases) when MDCL increases (decreases).

We further compare the offline and online testing results for individual scenarios. However, since MAE and MDCL are different metrics, we cannot directly compare them. Instead, we set threshold values for MAE and MDCL to translate these metrics into binary results (i.e., *acceptable* versus *unacceptable*) that can be compared. In particular, we interpret the online testing results of DNN *d* for a test scenario **s** as acceptable if *MDCL*(*d*,**s**) < 0.7 and unacceptable otherwise. Note that we have *MDCL*(*d*,**s**) < 0.7 when the departure from the centre of the lane observed during the simulation of **s** is less than around one meter. Based on domain expert knowledge, such a departure can be considered safe. We then compute a threshold value for MAE that is semantically similar to the 0.7 threshold for MDCL. To do so, we calculate the steering angle error that leads to the vehicle deviating from the centre of the lane by one meter. This, however, depends on the vehicle speed and the time it takes for the vehicle to reach such deviation. We assume the speed of the vehicle to be 30 km/h (i.e., the slowest vehicle speed when the vehicle is driving on normal roads) and the time required to depart from the centre of the lane to be 2.7 seconds (which is a conservative driver reaction time for braking (McGehee et al. 2000)). Given these assumptions, we compute the steering angle error corresponding to a one meter departure to be around 2.5^∘^. Thus, we consider the offline testing results of *d* for **s** as acceptable if *MAE*(*d*,*sim*(**s**)) < 0.1 (meaning the average prediction error is less than 2.5^∘^) and unacceptable otherwise.

#### Results

**Fig. 9:**
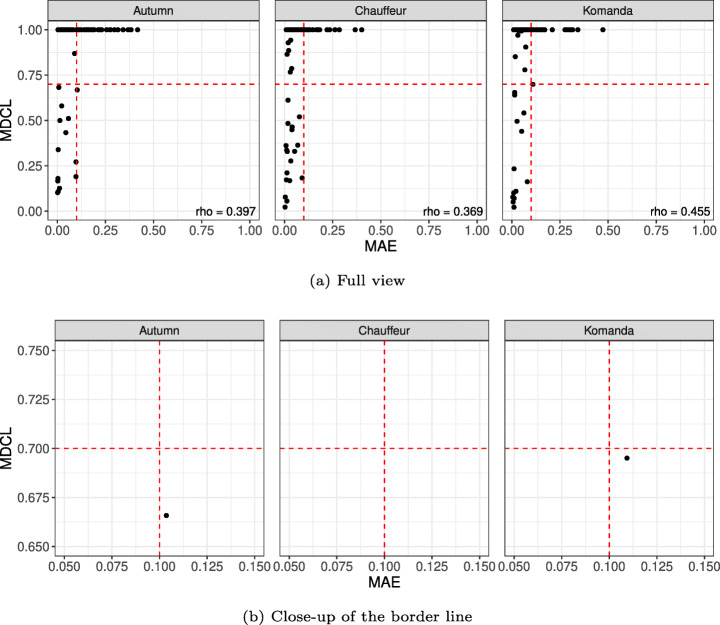
Comparison between offline and online testing results for all scenarios

Figure 9 shows the comparison between offline and online testing results in terms of MAE and MDCL values for all the randomly generated scenarios in $S^{\prime }$ where $|S^{\prime }| = 90$. We generated 90 scenarios because it is the number of scenarios required to achieve 2-way combinatorial coverage^4^ for all the attributes in our extended domain model. The x-axis is MAE (offline testing) and the y-axis is MDCL (online testing). The dashed lines represent the thresholds, i.e., 0.1 for MAE and 0.7 for MDCL. In the bottom-right corner of each diagram in Fig. 9, we show the Spearman correlation coefficients (*ρ*) between MAE and MDCL. For our three DNN models, *ρ* is not zero but less than 0.5, meaning that there are weak correlations between MAE and MDCL.

In Table 2, we have the number of scenarios classified by the offline and online testing results based on the thresholds. The results show that offline testing and online testing are not in agreement for 45.5%, 63.3%, and 60.0% of the 90 randomly generated scenarios for Autumn, Chauffeur, and Komanda, respectively. Surprisingly, we have only two cases (one from Autumn and one from Komanda) where the online testing result is acceptable while the offline testing result is not, and even these two exceptional cases are very close to the border line as shown in Fig. 9(b), i.e., (0.104,0.667) for Autumn and (0.109,0.695) for Komanda where (*x*,*y*) indicates MAE=*x* and MDCL=*y*. After analyzing the online testing results of these two cases in more detail, we found that MDCL was less than the threshold simply because the road was short, and would have been larger had the road been longer. Consequently, the results show that offline testing is significantly more optimistic than online testing for the disagreement scenarios.

**Table 2:** Number of scenarios classified by offline and online testing results

	MAE < 0.1	MAE ≥ 0.1	Total
a Autumn			
MDCL < 0.7	13	1	14
MDCL ≥ 0.7	40	36	76
Total	53	37	90
b Chauffeur			
MDCL < 0.7	18	0	18
MDCL ≥ 0.7	57	15	72
Total	75	15	90
c Komanda			
MDCL < 0.7	13	1	14
MDCL ≥ 0.7	53	23	76
Total	66	24	90

**Fig. 10:**
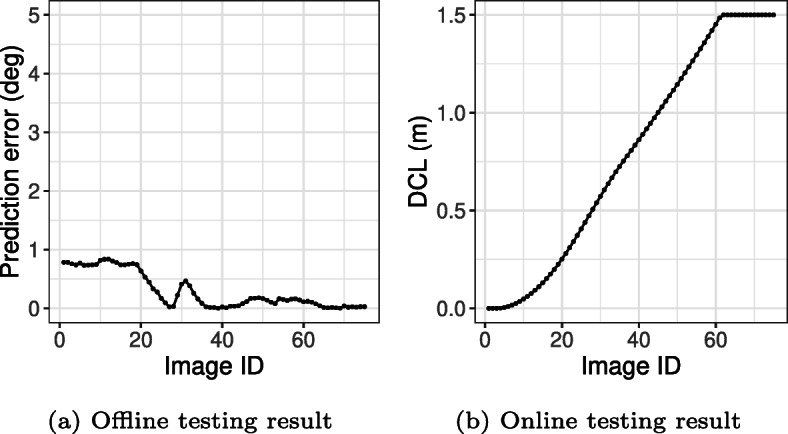
Example inconsistent results between offline and online testing

Figure 10 shows one of the scenarios on which offline and online testing disagreed. As shown in Fig. 10(a), the prediction error of the DNN for each image is always less than 1^∘^. This means that the DNN appears to be accurate enough according to offline testing. However, based on the online testing result in Fig. 10(b), the ego vehicle departs from the center of the lane in a critical way (i.e., more that 1.5 m). This is because, over time, small prediction errors accumulate, eventually causing a critical lane departure. Such accumulation of errors over time is only observable in online testing, and this also explains why there is no case where the online testing result is acceptable while the offline testing result is not. 
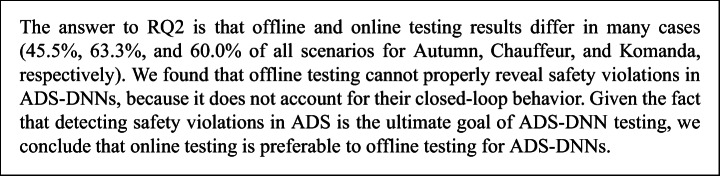

### RQ3: Rule Extraction

#### Setup

In RQ2, we showed that, for ADS-DNNs, offline prediction errors are not correlated with unsafe deviations observed during online testing. In other words, offline testing will not reveal some of the safety violations that can be revealed via online testing. However, offline and online testing are both essential steps in development and verification of DNNs (Zhang et al. 2020). A typical workflow for DNN testing is to first apply offline testing, which is a standard Machine Learning process, and then move to online testing, which is more expensive and requires engineers to invest significant time on integrating the DNN into a simulated application environment. The goal of this research question is to provide guidelines on how to combine offline and online testing results to increase the effectiveness of our overall testing approach (i.e., to reveal the most faults) while reducing testing cost. To achieve this goal, we identify conditions specified in terms of our domain model attributes (Fig. 3) that characterize when offline and online testing agree and when they disagree. We seek to derive these conditions for different DNN subjects. Provided with these conditions, we can identify testing scenarios that should be the focus of online testing, i.e., scenarios for which offline testing is ineffective, but online testing may reveal a safety violation.

In our work, as discussed in Section 2.3, each test scenario is specified as a vector of values assigned to the attributes in our domain model. By applying offline and online testing to all test scenario vectors, we can determine if it belongs to the category where offline and online testing results are in agreement or not. Using test vectors and their corresponding categories as a set of labelled data instances, we can then generate classification rules by applying well-known rule mining algorithms, such as RIPPER (Cohen 1995), to learn conditions on domain model attributes that lead to agreement or disagreement of offline and online testing results. To be able to learn these conditions with a high degree of accuracy, however, we need to gather a large collection of labelled data instances including test vectors ideally covering all combinations of value assignments to the attributes of our domain model. However, the number of all combinations of all the attribute values is more than 2^32^ since we have 32 attributes of enumeration types in our domain model that can take more than two values. Furthermore, the simulation time on a desktop with a 3.6 GHz Intel i9-9900k processor with 32 GB memory and graphic card Nvidia GeForce RTX 2080Ti is up to 20-30 minutes for each scenario depending upon attributes like road length and speed of ego vehicle. Therefore, it is impossible to cover all the combinations of value assignments to our domain model attributes.

To be able to learn conditions characterizing agreement and disagreement between offline and online testing in an effective and efficient way, we propose a three-step heuristic approach that focuses on learning rules with statistically high confidence while minimizing the number of test vectors (i.e., value assignments to domain model attributes) required for learning. Specifically, we incrementally generate new test vectors to be labelled by focusing on specific attributes to minimize the amount of data needed for classification while increasing statistical significance.

**Fig. 11:**
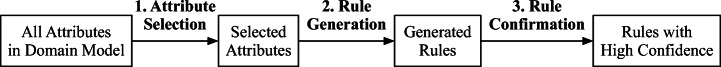
Overall workflow of the three-step heuristic approach to extract rules

Figure 11 outlines the workflow of the approach. The first step is *attribute selection*, which aims to reduce the search space by identifying a subset of attributes correlated with the differences between offline and online testing results for a DNN. The second step (*rule generation*) aims at extracting rules, for a given DNN, based on the selected attributes. The third step, *rule confirmation*, seeks to improve the statistical confidence of the extracted rules. In each of the steps, a minimal number of new data instances are incrementally generated. The details of the steps are described next:

1. *Attribute Selection*: In data mining, attribute selection (a.k.a., feature selection) strategies often rely on generating a large number of labelled data instances randomly to ensure data diversity and the uniformity of their distribution in the search space. Since labelling data instances (i.e., test vectors) in our work is expensive, we are limited regarding how many test vectors we can generate and label for the purpose of attribute selection. Therefore, instead of using a pure random strategy, as the input for our attribute selection strategy, we use *n*-way combinatorial testing to generate a relatively small number of diverse test vectors. The value of *n* is determined based on our time budget for labelling data (running test vectors in our work) and the number of attributes in our domain. In our work, for the purpose of attribute selection, we set *n* = 2. This led to generating 90 test vectors to be able to cover the pairwise combination of values of the 32 attributes in our domain model. For each test vector, we determine whether offline and online testing results are in agreement or not, resulting in the binary classification of our test vectors. We then perform the attribute selection using the Random Forest algorithm (Genuer et al. 2010). We use the concept of variable importance in Random Forests to select important attributes in our domain model since it has been reported to be accurate in general (Archer and Kimes 2008; Genuer et al. 2010). Table 3 shows the selected attributes for each DNN. Six, four, and two attributes are selected for Autumn, Chauffeur, and Komanda, respectively.
2. *Rule Generation*: At this step, we trim the set of test vectors generated in the attribute selection step by hiding, from each vector, the attributes that were not selected in the previous step. The result is a set of labelled test vectors that only include values for the important attributes of our domain (i.e., those attributes selected in the previous step). This set, however, does not necessarily cover different combinations of the values for the selected attributes. Hence, we enhance this set by generating a number of new test vectors. We use *n*-way combinatorial test generation again, but this time we only consider the attributes selected in the previous step in the test generation (i.e., the attributes that were not selected in step one are simply set a random value). For this step, we choose *n* = 3 since we are dealing with a smaller number attributes and are able to generate more value combinations during the same test time budget. For Komanda, we use all combinations since we have only two selected attributes. The number of new test vectors therefore varies depending on the selected attributes for each DNN. The new test vectors, together with the 90 test vectors generated in the previous step, are used for rule generation. We extract rules using the RIPPER algorithm (Cohen 1995), yielding a set of rules for our DNN under analysis. Each generated rule is a tuple (*if*,*label*) where *if* describes conditions on the values of the selected attributes (e.g., *Vehicle.speed*> 10 ∧ *Road.type* = *Curved*) and *label* describes a class (i.e., *agree* or *disagree*). We can estimate the accuracy of each rule as the number of test vectors labelled by *label* and satisfying the *if* conditions over the number of test vectors satisfying *if*. Table 4 shows the rules generated for each DNN. As shown in the table, we obtain three rules for Autumn, four rules for Chauffeur, and two rules for Komanda. Each rule has an *if* part, described as a conjunction of predicates defined over our domain model attributes, and a *label* part that can be either *agree* or *disagree*. For the accuracy values, we also report the 95% Confidence Interval (CI). For example, for the second rule of Autumn, the accuracy value 0.88 ± 0.20 means that the true accuracy value has a 95% probability of being in the interval [0.68 1.00]. The CI range is quite large since, in our work, we have minimized the total number of test vectors, and therefore the number of test vectors satisfying the conditions *if* for each rule can be small. Hence we may not be able establish reasonably narrow CIs for the accuracy values of the generated rules. To alleviate this issue, we use a third step to increase the statistical confidence in the estimated accuracy of the generated rules.
3. *Rule Confirmation*: The basic idea is to reduce the CI length by providing more data instances for each rule. For each rule, we repeatedly generate a data instance satisfying the *if* part of the rule until the CI length of the estimated accuracy of the rule with a 95% confidence level is less than a threshold *λ*. In our experiments, we set *λ* = 0.2, meaning ± 0.1. Note that we generate new data instances only for the extracted rules to efficiently reduce the CI length of the rules. The final results after performing the Rule Confirmation step is shown in Table 5 and will be discussed in the next section (Section 3.4.2).

**Table 3:** Intermediate Results: Selected Attributes

DNN	Selected Attributes
Autumn	Road.type, Road.laneLineColor, Road.curbLinePattern,
	Vehicle.laneNumber, Vehicle.headLights, Weather.condition
Chauffeur	Road.type, Road.roadSpecificProperty, Vehicle.fogLights,
	Environment.underlay
Komanda	Weather.type, Environment.bulidings

**Table 4:** Intermediate Results: Generated Rules

DNN	Rule	Accuracy	
Autumn	If *Road.curbLanePattern* = *Dashed* then *disagree*	0.58	± 0.11
	If *Road.type* = *Curved* then *disagree*	0.88	± 0.20
	Other than mentioned above then *agree*	0.65	± 0.11
Chauffeur	If *Vehicle.fogLights* = *True*, then *agree*	0.59	± 0.13
	If *Road.type* = *Straight* ∧ *Environment.underlay* = *Pavement* then *agree*	0.90	± 0.18
	If *Road.type* = *Curved* then *agree*	0.70	± 0.28
	Other than mentioned above then *disagree*	0.76	± 0.09
Komanda	If *Weather.type* = *Rainy* ∧ *Environment.buildings* = *False* then *agree*	0.76	± 0.18
	Other than mentioned above then *disagree*	0.69	± 0.11

**Table 5:** Rule Extraction Results

DNN	ID	Rule	Accuracy	
Autumn	A1	If *Road.curbLanePattern* = *Dashed* then *disagree*	0.61	± 0.10
	A2	If *Road.type* = *Curved* then *disagree*	0.95	± 0.10
	A3	Other than mentioned above then *agree*	0.61	± 0.10
Chauffeur	C1	If *Vehicle.fogLights* = *True*, then *agree*	0.58	± 0.10
	C2	If *Road.type* = *Straight* ∧ *Environment.underlay* = *Pavement* then *agree*	0.71	± 0.10
	C3	If *Road.type* = *Curved* then *agree*	0.55	± 0.10
	C4	Other than mentioned above then *disagree*	0.76	± 0.09
Komanda	K1	If *Weather.type* = *Rainy* ∧ *Environment.buildings* = *False* then *agree*	0.59	± 0.10
	K2	Other than mentioned above then *disagree*	0.67	± 0.10

#### Results

Table 5 shows the rules generated for our three DNN subjects after applying the process described in Section 3.4.1 and Fig. 11. Note that in contrast to the results reported in Table 4, the accuracy values in Table 5 are those obtained after applying the rule confirmation step. For example, for the first rule for Autumn, the accuracy of 0.61 ± 0.10 means that around 61% of the scenarios that satisfy the condition *Road.curbLanePattern* = *Dashed* are labelled with *disagree*. Thanks to our rule confirmation step, we are able to ascertain the accuracy levels of rules within a narrower 95% confidence interval. In our work, the rule accuracy indicates the predictive power of the rule. For example, the second rule for Autumn is highly accurate and hence predictive (more than 85% of scenarios). Hereafter, for simplicity, we use the IDs indicated in Table 5 to refer to the rules.

Overall, there is no rule predicting *agree* with an accuracy above 0.71. This means that there is no condition with accuracy above 0.71 where offline testing results conform to online testing results. That is, the test results for scenarios that match the “agree” rule conditions in Table 5 may still differ during offline and online testing with a high probability. Therefore, based on our results, we are not able to identify conditions that can characterize, with a high accuracy, agreement between offline and online testing to help lift offline testing results to online testing and reduce the amount of online testing needed. Our results, further, suggest that, at least for ADS-DNNs, we may not be able to find rules that can, in general, differentiate between offline and online testing behaviors. As can be seen from the table, there is not much similarity between the rules we have obtained for different DNNs. This is because these DNNs have different architectures, use different features of the input images for prediction and are trained differently.

However, our observations show that these rules still may provide valuable insights as to how different DNNs work. When an attribute appears in a rule, it indicates that the attribute has a significant impact on the DNN output, and hence, this attribute can be used to classify both the situations where offline testing is as good as online testing (i.e., DNN prediction errors indeed indicate a safety violation) as well as the dual situations where offline testing is simply too optimistic. For example, the attributes *Weather.type* and *Environment.buildings* appear only in the conditions for the rules of Komanda. On the other hand, for Chauffeur and Autumn, the attributes appearing in the rule conditions are related to the road shape and the road lane patterns. This confirms the fact that Komanda uses full images to predict steering angles while the Chauffeur and Autumn focus on the road-side views in the images, as noted in Section 3.1.

Another reason explaining differences across DNNs is that some DNNs are inaccurate for certain attributes regardless of testing modes, which means that both offline testing and online testing are capable of detecting the faulty behaviors of these DNNs with a relatively high probability. For example, we found that Chauffeur is, in general, inaccurate for predicting steering angles for curved roads. Due to this weakness, both offline and online testing results are in agreement when *Road.type* is *Curved*, as shown in C3.

The last reason for differences is that, as shown in RQ1, Chauffeur works relatively better on real-world images than on simulated images. Since Chauffeur is not effective with simulated images, it may yield more prediction errors in offline testing, and hence, offline and online testing results are more likely to be in agreement as “unacceptable”. This explains why we have three “agree” rules, namely C1, C2 and C3, for Chauffeur while for other DNNs we have fewer “agree” rules. 
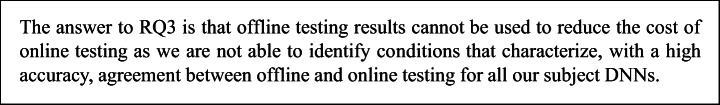


### Threats to Validity

In RQ1, we propose a two-step approach that builds simulator-generated datasets comparable to a given real-life dataset. While it achieves its objective, as shown in Section 3.2.2, the simulated images are still different from the real images. However, we confirmed that the prediction errors obtained by applying our subject DNNs to the simulator-generated datasets are comparable with those obtained for their corresponding real-life datasets. Thus, the conclusion that offline and online testing results often disagree with each other is valid.

We used a few thresholds that may affect the experimental results in RQ2 and RQ3. To reduce the chances of misinterpreting the results, we selected intuitive and physically interpretable metrics to evaluate both offline and online test results (i.e, prediction errors and safety violations), and defined threshold values based on common sense and experience. Further, adopting different threshold values, as long as they are within a reasonable range, does not change our findings. For example, if we use *MAE*(*d*,*sim*(**s**)) < 0.05 as a threshold in offline testing results instead of *MAE*(*d*,*sim*(**s**)) < 0.1, the numbers of scenarios in Table 2 change. However, it does not change the correlation analysis results and the fact that we have many scenarios for which offline and online testing results disagree, nor does it change the conclusion that offline testing is more optimistic than online testing.

Different ADS-DNNs may lead to different results. For example, we may able to identify conditions that can characterize, with a high accuracy, agreement between offline and online testing results to lift offline testing results to online testing and reduce the amount of online testing needed for a specific ADS-DNN. To mitigate such a threat, we tried our best to find all candidate ADS-DNNs in the literature and selected the three subject ADS-DNNs (i.e., Autumn, Chauffeur, and Komanda) that are publicly available and sufficiently accurate for steering angle predictions.

Though we focused, in our case study, on only two lane-keeping DNNs (steering prediction)—which have rather simple structures and do not support braking or acceleration, our findings are applicable to all DNNs in an ADS context as long as the closed-loop behavior of the ADS matters.

## Discussion

### Online Testing using Simulators

One important purpose of online testing is to test a trained ADS-DNN with the newest unseen data that potentially appear in the application environments of the ADS-DNN. However, because simulators cannot express all the complexity and diversity of the real world, online testing using simulators cannot cover all possible scenarios in the real world. For example, in the case of online testing using a simulator that cannot express weather changes, certain safety violations of the ADS-DNN that occurs only in rainy weather cannot be found.

Nevertheless, considering the problems of online testing in the real world, especially the cost and risk, online testing using a simulator is inevitable. Indeed, according to our industrial partners in the automotive industry, due to the excessive amount of manpower and resources required to collect real-world data, it is impossible to gather sufficient and diverse real-world data. On the other hand, a simulator can generate sufficiently diverse data at a much lower cost and risk.

Furthermore, simulator-based test input generation has an additional advantage regarding the test oracle problem (Barr et al. 2015). When we use simulators for online testing, the generation of test oracles is completely automated. For real-life datasets, however, test oracles may need to be manually specified which is labor-intensive and time-consuming. For example, for ADS-DNNs, the driver’s maneuvers and the data gathered from the various sensors and cameras during online testing in the real world may not contain sufficient information to automatically generate test oracles. In contrast, simulators are able to generate labeled datasets, from which test oracles can be automated, for various controlling, sensing, and image recognition applications. But, as expected, the accuracy of test results and oracles depends on the fidelity of simulators.

### Offline vs. Online Testing: What to Use in Practice?

Experimental results show that offline testing cannot properly detect safety requirements violations identified in online testing. Offline testing is in fact inadequate to identify faulty behaviors for ADS-DNNs with closed-loop behavior. In other words, online testing is essential to adequately detect safety violations in ADS-DNNs, where interactions with the application environment are important. In particular, online testing using a simulator is highly recommended if a high-fidelity simulator is available.

However, online testing is not essential in all cases. When testing ADS-DNNs without closed-loop behavior, offline and online testing results are expected to be similar because errors are not accumulating over time. For example, in the case of an ADS-DNN that simply warns the driver instead of directly controlling the steering when necessary, there is no closed-loop since the DNN’s predictions do not actually control the vehicle, and therefore offline testing would be sufficient.

### Open Challenges

There are also challenges that need to be addressed in online testing. For example, the higher the fidelity of a simulator, the more time it takes to simulate, which has direct impact on the cost of online testing. In particular, when using a search-based technique, online testing may take a very long time because the simulator must be repeatedly executed for various scenarios. Therefore, more research is required to reduce the cost of online testing.

Research on high-fidelity simulators is also essential. As discussed in Section 4.1, online testing using a simulator cannot completely cover all possible scenarios in the real world. However, by utilizing a simulator higher fidelity, the risk of uncovered scenarios could be significantly reduced (Shah et al. 2018; Rong et al. 2020). Research on how to lower the risk through a more systematic approach is also needed.

## Related Work

Table 6 summarizes DNN testing approaches specifically proposed in the context of autonomous driving systems. Approaches to the general problem of testing machine learning systems are discussed in the recent survey by Zhang et al. (2020).

**Table 6:** Summary of DNN testing studies in the context of autonomous driving

Author(s)	Testing mode	DNN’s role	Summary
Dreossi et al. (2017)	Offline	Object detection	Test image generation by arranging basic objects using greedy search
Pei et al. (2017)	Offline	Lane keeping	Coverage-based label-preserving test image generation using joint optimization with gradient ascent
Codevilla et al. (2018)	Offline and online	Lane keeping	Improving the correlation between offline and online testing results by selecting an appropriate testing dataset and suitable offline metrics
Tian et al. (2018)	Offline	Lane keeping	Coverage-based label-preserving test image generation using greedy search with simple image transformations
Tuncali et al. (2018)	Online	Object detection	Test scenario generation using the combination of covering arrays and simulated annealing
Wicker et al. (2018)	Offline	Traffic sign recognition	Adversarial image generation using feature extraction
Zhang et al. (2018)	Offline	Lane keeping	Label-preserving test image generation using Generative Adversarial Networks (GANs)
Zhou et al. (2018)	Offline	Lane keeping	Adversarial billboard-image generation for digital and physical adversarial perturbation
Gambi et al. (2019)	Online	Lane keeping	Automatic virtual road network generation using search-based Procedural Content Generation (PCG)
Kim et al. (2019)	Offline	Lane keeping	Improving the accuracy of DNNs against adversarial examples using surprise adequacy
Majumdar et al. (2019)	Online	Object detection, lane keeping	Test scenario description language and simulation-based test scenario generation to cover parameterized environments
Zhou and Sun (2019)	Offline	Object detection	Combination of Metamorphic Testing (MT) and fuzzing for 3-dimensional point cloud data
This article	Offline and online	Lane keeping	Comparison between offline and online testing results and investigate if we can use offline testing results to run fewer tests during online testing

In Table 6, approaches for online testing are highlighted grey. As the table shows, most of existing approaches focus on the offline testing mode only, where DNNs are seen as individual units without accounting for the closed-loop behavior of a DNN-based ADS. Their goal is to generate test data (either images or 3-dimensional point clouds) that lead to DNN prediction errors. Dreossi et al. (2017) synthesized images for driving scenes by arranging basic objects (e.g., road backgrounds and vehicles) and tuning image parameters (e.g., brightness, contrast, and saturation). Pei et al. (2017) proposed DeepXplore, an approach that synthesizes images by solving a joint optimization problem that maximizes both neuron coverage (i.e., the rate of activated neurons) and differential behaviors of multiple DNNs for the synthesized images. Tian et al. (2018) presented DeepTest, an approach that generates label-preserving images from training data using greedy search for combining simple image transformations (e.g., rotate, scale, and for and rain effects) to increase neuron coverage. Wicker et al. (2018) generated adversarial examples, i.e., small perturbations that are almost imperceptible by humans but causing DNN misclassifications, using feature extraction from images. Zhang et al. (2018) presented DeepRoad, an approach that produces various driving scenes and weather conditions by applying Generative Adversarial Networks (GANs) along with corresponding real-world weather scenes. Zhou and Sun (2019) combined Metamorphic Testing (MT) and Fuzzing for 3-dimensional point cloud data generated by a LiDAR sensor to reveal erroneous behaviors of an object detection DNN. Zhou et al. (2018) proposed DeepBillboard, an approach that produces both digital and physical adversarial billboard images to continuously mislead the DNN across dashboard camera frames. While this work is different from the other offline testing studies as it introduces adversarial attacks through sequences of frames, its goal is still the generation of test images to reveal DNN prediction errors. In contrast, Kim et al. (2019) defined a coverage criterion, called *surprise adequacy*, based on the behavior of DNN-based systems with respect to their training data. Images generated by DeepTest were sampled to improve such coverage and used to increase the accuracy of the DNN against adversarial examples.

Online testing studies exercise the ADS closed-loop behavior and generate test driving scenarios that cause safety violations, such as unintended lane departure or collision with pedestrians. Tuncali et al. (2018) were the first to raise the problem that previous works mostly focused on the DNNs, without accounting for the closed-loop behavior of the system. Gambi et al. (2019) also pointed out that testing DNNs for ADS using only single frames cannot be used to evaluate closed-loop properties of ADS. They presented AsFault, a tool that generates virtual roads which cause self-driving cars to depart from their lane. Majumdar et al. (2019) presented a language for describing test driving scenarios in a parametric way and provided Paracosm, a simulation-based testing tool that generates a set of test parameters in such a way as to achieve diversity. We should note that all the online testing studies rely on virtual (simulated) environments, since, as mentioned before, testing DNNs for ADS in real traffic is dangerous and expensive. Further, there is a growing body of evidence indicating that simulation-based testing is effective at finding violations. For example, recent studies for robotic applications show that simulation-based testing of robot function models not only reveals most bugs identified during outdoor robot testing, but that it can additionally reveal several bugs that could not have been detected by outdoor testing (Sotiropoulos et al. 2017).

There is only one study comparing offline and online testing results by investigating the correlations between offline and online testing prediction error metrics (Codevilla et al. 2018). The authors found that the correlation between offline prediction and online performance is weak, which is consistent with the results of this article. They also found two ways for improving the correlations: (1) augmenting the testing data (e.g., include images from three cameras, i.e., a forward-facing one and two lateral cameras facing 30 degrees left and right, instead of having images from one forward-facing camera) and (2) selecting a proper offline testing metric (e.g., Mean Absolute Error other than Mean Squared Error). Their analysis relies on the offline and online testing of DNNs trained by simulator-generated images, while our DNNs are trained with real-world images. Nevertheless, consistent with our results, they concluded that offline testing is not adequate. Furthermore, beyond simple correlations and in order to draw more actionable conclusions, our investigation looked at whether offline testing was a sufficiently reliable mechanism for detecting safety violations in comparison to online testing. Last, we investigated whether offline and online testing results could agree under certain conditions, so as to take advantage of the lower cost of offline testing in such situations.

## Conclusion

This article presents a comprehensive case study to compare two distinct testing phases of Deep Neural Networks (DNNs), namely offline testing and online testing, in the context of Automated Driving Systems (ADS). Offline testing evaluates DNN prediction errors based on test data that are generated without involving the DNN under test. In contrast, online testing determines safety requirement violations of a DNN-based system in a specific application environment based on test data generated dynamically from interactions between the DNN under test and its environment. We aimed to determine *how offline and online testing results differ or complement each other* and *if we can exploit offline testing results to run fewer tests during online testing to reduce the testing cost*. We additionally investigated if we can use simulator-generated datasets as a reliable substitute to real-world datasets for DNN testing.

The experimental results on the three best performing ADS-DNNs from the Udacity Self-Driving Car Challenge 2 (Udacity 2016a) show that simulator-generated datasets yield DNN prediction errors that are similar to those obtained by testing DNNs with real-world datasets. Also, offline testing is more optimistic than online testing as many safety violations identified by online testing could not be identified by offline testing, while large prediction errors generated by offline testing always led to severe safety violations detectable by online testing. Furthermore, the experimental results show that we cannot exploit offline testing results to reduce the cost of online testing in practice since we are not able to identify specific situations where offline testing could be as accurate as online testing in identifying safety violations.

The results of this paper have important practical implications for DNN testing, not only in an ADS context but also in other CPS where the closed-loop behavior of DNNs matters. Specifically, both researchers and practitioners should focus more on online testing as offline testing is not able to properly determine safety requirement violations of the DNN-based systems under test.

Considering the expensive cost of online testing, even using a high-fidelity simulator instead of a real-world environment, our results also call for more efficient online testing approaches. As part of future work, we plan to develop an approach for automatic test scenario generation using surrogate models and search-based testing to efficiently identify safety critical test scenarios for online testing.

## Footnotes

1 This is how Tian et al. (2018) have interpreted the steering angle values provided along with the Udacity dataset, and we follow their interpretation. We were not able to find any explicit information about the measurement unit of these values anywhere else.

2 Autumn’s RMSE is not presented in the final leaderboard.

3 If *f* has multiple points of the minima, one of them is randomly returned.

4 We use PICT (https://github.com/microsoft/pict) to compute combinatorial coverage.
